# Outcomes by cardiovascular magnetic resonance in patients with clinically suspected myocardial inflammation

**DOI:** 10.1007/s10554-026-03711-y

**Published:** 2026-04-18

**Authors:** Eike Nagel, Gerry Carr-White, Andreas Rolf, Hafisyatul Zainal, Moises Vasquez, Hui Zhou, Luca Arcari, Faraz Pathan, Silvia Valbuena, Rocio Hinojar, Eleftherios Vidalakis, Michael Kolentinis, Tommaso D’Angelo, Elen Sahara, M. Ludovica Carerj, Julia Treiber, Michael Marber, Till Keller, Valentina O. Puntmann

**Affiliations:** 1 Institute of Experimental and Translational Cardiovascular Imaging, Goethe University Frankfurt, Frankfurt am Main, Germany; 2 DZHK Centre for Cardiovascular Imaging, partner site Rhein-Main, Goethe University, Frankfurt, Germany; 3 Department of Cardiology, Guy’s and St Thomas Hospital, London, UK; 4 Department of Cardiology, Kerckhoff Hospital, University Giessen, Bad Nauheim, Germany; 5 King’s College London, Cardiovascular Sciences, London, UK

**Keywords:** Myocardial inflammation, Diagnosis, Cardiovascular magnetic resonance, T2 mapping

## Abstract

Myocardial inflammation is a key driver of adverse outcomes; yet its clinical recognition remains challenging because of heterogeneous presentation and non-standardised prognostic frameworks. While prior studies linked troponin and late gadolinium enhancement (LGE) by cardiovascular magnetic resonance imaging (CMR) to outcomes, the relative prognostic value of individual non-invasive markers has not been systematically evaluated. We systematically evaluated the prognostic value of biomarkers and non-invasive imaging variables in patients with clinically suspected myocardial inflammation presenting to tertiary centres and referred for CMR diagnostic evaluation. The primary endpoint was major adverse cardiovascular events (MACE). A total of 722 patients (median age: 50 years [IQR: 40–61]; 422 males [58%]) were included. Over a median follow-up of 19 months (IQR: 15–23), 64 patients (9%) experienced MACE. In univariable analyses, age, haematocrit, C-reactive protein, high-sensitivity troponin T (hs-TropT), ventricular function, cardiac volumes and mass, late gadolinium enhancement (LGE), and native T1 and T2 values were associated with the endpoint. In multivariable Cox regression, native T2, hs-TropT, and LGE were independently predictive of MACE (model χ² = 81.64, *p* < 0.001). Patients with high-grade myocardial inflammation had the highest mid-term event risk (*p* < 0.001). Among patients with negative troponin, T2 and left ventricular end-diastolic volume (LVEDV), but not LGE, were predictive of MACE. In patients with clinically suspected myocardial inflammation, standardised CMR-based markers provide independent and incremental prognostic information, supporting their role in improved risk stratification beyond composite criteria.

**Supplementary Information:**

The online version contains supplementary material available at 10.1007/s10554-026-03711-y.

## Introduction

Myocardial inflammation is a prominent cause of adverse cardiovascular outcomes, including non-ischaemic cardiomyopathy (NICM), heart failure (HF) and sudden cardiac death [1]. However, clinical recognition of myocarditis remains challenging because of its heterogeneous presentation and the lack of standardized diagnostic criteria with consistent accuracy [2, 3]. Currently, non-invasive tests for myocardial inflammation target distinct pathological features: *myocardial injury* detected by troponin, *non-ischaemic scarring* by late gadolinium enhancement (LGE) by cardiovascular magnetic resonance (CMR), and *myocardial oedema* by T1 and T2 imaging [4, 5]. When measured within the detectable time-window, high-sensitivity troponin is often elevated in patients with suspected myocarditis [2, 6–9], especially in infarct-like myocarditis, a clinical presentation mimicking acute coronary syndrome (central chest pain, ST-segment changes and troponin rise), but without obstructive coronary artery disease. In this clinical context, subepicardial or regional non-ischaemic LGE denotes regional necrotic injury following acute viral infection [2, 10]. However, prognostic role of troponin in myocardial inflammation remains largely unclear [3, 11]. Due to higher sensitivity compared with conventional T2 weighted imaging [12, 13], myocardial mapping techniques have gained traction and are increasingly incorporated into clinical practice [14, 15] Click or tap here to enter text. Myocardial mapping methods quantify tissue characteristics based on magnetization relaxation rates. In myocardial inflammation, T2 mapping primarily reflects tissue water content, while T1 mapping captures both oedema and interstitial tissue expansion [16–18]. Studies across the spectrum of non-ischaemic cardiomyopathies have demonstrated the high diagnostic accuracy and prognostic value of mapping measures, supporting their inclusion into practice guidelines [1, 14, 15, 19].

Despite these advances, the relative prognostic contributions of individual non-invasive tests in myocardial inflammation have not been comprehensively assessed. Prior CMR studies in viral myocarditis linked LGE to cardiac mortality and HF [20–22], whereas a recent investigation using composite diagnostic criteria of conventional and novel techniques reported limited overall prognostic agreement in clinically suspected myocardial inflammation [3]. The objective of the present study was therefore to systematically evaluate the prognostic significance of non-invasive biomarkers and imaging parameters in patients with clinically suspected myocardial inflammation undergoing standardised CMR as part of routine evaluation.

## Methods

**Fig. 1:**
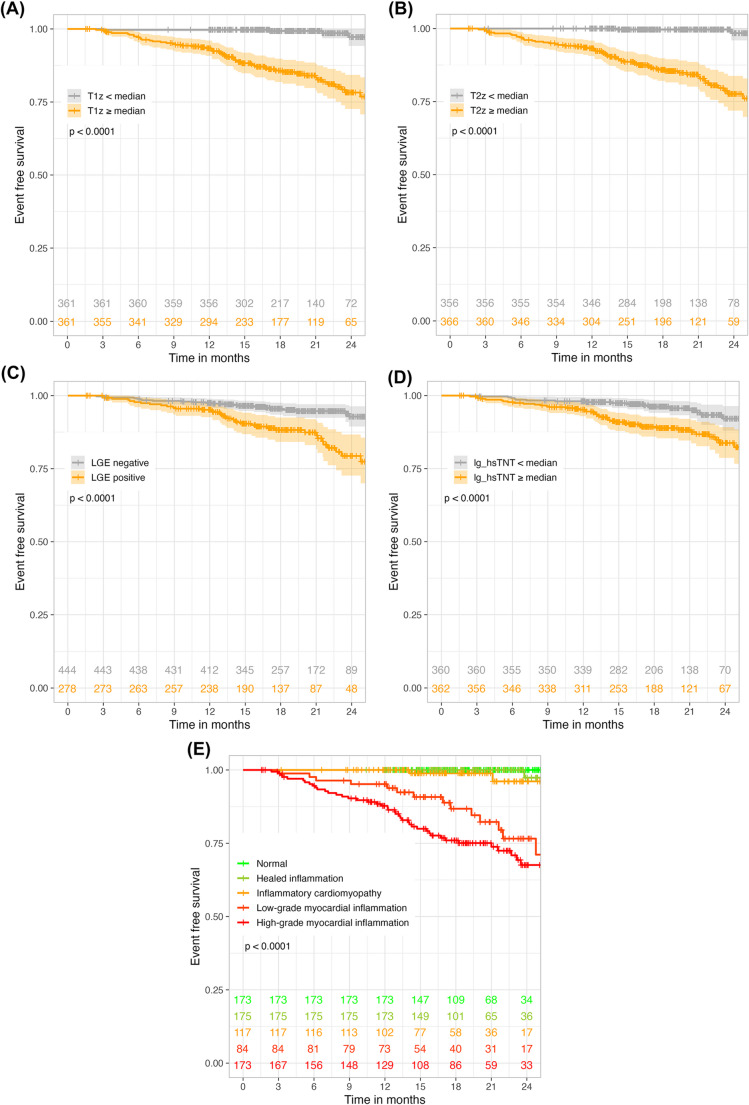
Kaplan–Meier curves for MACE according to individual measures: native T2 (χ² = 63.4), LGE (χ² = 22.11), hs-TropT (χ² = 15.94), and the CMR-based definition of myocardial inflammation (χ² = 115.9). All comparisons were statistically significant (*p* < 0.001). **A**. Native T1 ((χ² 56.38, *p* < 0.0001). **B**. Native T2 ((χ² 63.4, *p* < 0.0001). **C**. LGE ((χ² 22.11, *p* < 0.0001). **D**. High-sensitive Troponin (χ² 15.94, *p* < 0.0001). **E**. CMR-based definition of myocardial inflammation (χ² = 115.9, *p* < 0.0001)

This was a prospective, longitudinal, multicentre observational study of consecutive adult patients referred to CMR for diagnostic evaluation. The study cohort was recruited between October 2011 and December 2019 at three centres using standardised CMR protocols: University Hospital Frankfurt, Kerckhoff Clinic Bad Nauheim (both in Germany), and Guy’s and St Thomas’s Hospital, London (United Kingdom). Patient inclusion is summarised in STROBE diagram (Fig. 1S, Supplementary material). All procedures were carried out in accordance with the Declaration of Helsinki (2013) and relevant clinical guidelines [4]. The study protocol was reviewed and approved by respective institutional ethics committees (The Ethics Committee of Guys and St Thomas’ Hospital, NCT02407197, and the Ethics Committee of University Hospital Frankfurt, NCT03749343). Written informed consent was obtained from all participants**.**

Eligible patients were adults (≥ 18 years) with no prior major adverse cardiovascular events (MACE). Clinically suspected myocardial inflammation was diagnosed by attending physicians according to contemporary guideline-based criteria, requiring cardiac symptoms in combination with history of flu-like illness, electrocardiographic abnormalities, elevated inflammatory markers and/or troponin levels, and the absence of obstructive coronary artery disease [4]. Key exclusion criteria included pre-existing cardiac conditions (ischaemic heart disease by invasive coronary angiography, cardiac CT or CMR with myocardial perfusion, or specific cardiomyopathies), prior heart transplantation, prior cardio-oncological treatment, systemic inflammatory conditions, chronic infections, possible uremic cardiomyopathy (defined by left ventricular (LV) dysfunction accompanied by estimated glomerular filtration rate (eGFR) of less than 30 mL/min/1.73m2) [23], and contraindications to contrast-enhanced CMR, including pregnancy or MRI-unsafe devices. Baseline data collection included demographics, symptoms, results of clinical blood tests (hs-TropT) and CMR measurements. Hs-TropT values were interpreted using a 99th percentile cutoff of 13.9 ng/L to define abnormal levels.

All CMR studies were performed on 3.0 Tesla clinical scanners (Skyra, Siemens Healthineers, in Frankfurt and Bad Nauheim, Germany, software version VE11; Philips Achieva in London (software version pre-Upgrade5), using standardised imaging protocols and harmonised sequence parameters, as previously reported [20, 21]. Conventional sequences were used to acquire cardiac function, volumes, mass and scar imaging. Myocardial T1 and T2 mapping were performed in a single midventricular short-axis slice using validated research sequences. T1 mapping employed a modified Look-Locker inversion recovery sequence. T2 values were obtained using T2-FLASH [24] (on Siemens platform), and T2-GraSE (Philips platform) sequences [25]. LGE imaging was acquired approximately 10 min after administration of 0.1 mmol/kg body weight of gadobutrol (Gadovist^®^, Bayer AG, Leverkusen, Germany). Detailed sequence parameters are provided in the Supplementary Material. Image analysis was conducted centrally by the core laboratory following standardised operating procedures. All datasets were pseudonymized, and analysts were blinded to clinical information. CMR acquisition followed standardised protocols at each site; image analysis was performed centrally at the core laboratory. Clinical testing, including biomarker sampling and treatment decisions, followed local routine care. Cardiac volumes, function, and mass were quantified using semi-automated contour detection (SuiteHeart^®^, Neosoft, Pewaukee, WI). Myocardial perfusion and LGE images were interpreted according to standardised post-processing guidelines. LGE was defined visually by an increase in signal intensity in at least two orthogonal acquisitions and categorised by its predominant pattern as ischaemic or non-ischaemic.

T1 and T2 relaxation times were measured conservatively within the septal myocardium of the midventricular short-axis slice, with motion-correction applied [26]. Areas of LGE were excluded from regions of interest to avoid confounding between diffuse fibrosis and focal scar. The CMR definitions of myocardial inflammatory involvement have been published previously [2] are provided in Supplementary material using sequence specific cut-off values: high-grade inflammation (T1 ≥ 5SD, T2 ≥ 2SD), low-grade (T1 ≥ 2SD, T2 ≥ 2SD); cardiomyopathy (diffuse fibrosis; T1 ≥ 2SD, T2 < 2SD); healed myocarditis (T1 < 2SD, T2 < 2SD, presence of non-ischemic LGE); normal (T1 < 2SD, T2 < 2SD, no evidence of myocardial impairment, *restitutio ad integrum*).

Patient follow-up was conducted during routine outpatient visits or through structured telephone interviews in 12 months intervals. The cause of death was ascertained by review of medical records, death certificates, autopsy findings, or interviews with family members or witnesses. Event adjudication was performed independently by experienced physicians, who were blinded to the study team. Deaths were classified according to a modified Hinkle-Thaler system. The primary outcome was a composite MACE, defined as cardiovascular death, SCD, or appropriate implantable cardioverter-defibrillator (ICD) therapy. Only the first qualifying event for each patient, counted from the date of written consent, was included in the analyses. Blood biomarkers were obtained at clinical presentation as part of routine admission workup; CMR was performed as part of the diagnostic evaluation pathway. Event definitions [27] are detailed in the Supplementary material.

Statistical analyses were performed using SPSS version 25.0 (SPSS Inc., Chicago, IL, USA) and RStudio version 1.2.5001 (RStudio Inc.), utilizing the following R packages: ‘rms’, ‘survival’, ‘survminer’, ’caret’, ‘nricens’, ‘mice’. Data are presented as frequencies (percentages) for categorical variables and for continuous variables. Continuous variables were assessed for normality using the Shapiro–Wilk test. Normally distributed variables are reported as means (standard deviations, SD), non-normally distributed variables as median [IQR] and were log-transformed prior to Cox modelling. Between-group comparisons were performed using the Mann–Whitney U test for continuous variables and Fisher’s exact test for categorical variables. Univariable Cox proportional-hazards models assessed associations between baseline variables and outcomes, with results reported as hazard ratios (HRs) and 95% confidence intervals (CIs).

Multivariable model selection was performed using stepwise regression based on the Akaike Information Criterion (AIC), allowing bidirectional variable selection to identify the most parsimonious model while assessing multicollinearity. Candidate variables were defined a priori based on clinical relevance and univariable associations, and the number of predictors was limited according to the number of events to avoid overfitting. With 64 primary endpoints and a candidate set of six to seven predictors, the events-per-variable ratio was approximately 9, satisfying the accepted minimum threshold for stable Cox regression. Age and systolic blood pressure were included as candidate covariates given observed baseline imbalances. Non-normally distributed variables were log-transformed before inclusion in the model. T1 and T2 values were normalised using sequence-specific z-scores. Kaplan–Meier curves were used to illustrate time-to-event relationships. Missing data were imputed using predictive mean matching, and imputation performance was assessed for potential bias. All statistical tests were two-tailed, and a p-value < 0.05 was considered statistically significant.

## Results

Baseline characteristics of the study cohort are summarised in Table 1. In total, 722 patients were included in the final analysis (median age 50 years [IQR 40–61]; 422 were male [58%]). Six patients were excluded from analysis due to contraindications to CMR (*n* = 3) or non-diagnostic image quality (*n* = 3). Missing data were present in 116 patients (16%), most frequently affecting NYHA class (*n* = 109, 15%) and hs-CRP (*n* = 79, 10%).

**Table 1:** Subjects’ characteristics. Baseline clinical characteristics stratified by MACE status. BP, blood pressure; BMI, body mass index; NYHA, New York Heart Association; eGFR, estimated glomerular filtration rate; hs-CRP, high-sensitivity C-reactive protein; hs-TropT, high-sensitivity troponin T; log₁₀, log-transformed; LV, left ventricular; EDV, end-diastolic volume; ESV, end-systolic volume; EF, ejection fraction; LA, left atrium; LGE, late gadolinium enhancement; z, z-score normalisation. Values are expressed as median (IQR) or counts (%)

Variable	No MACE (*n* = 658)	MACE (*n* = 64)	Sig. (*p*-value)
Age (years)	49.6 ± 15.5	54.5 ± 15.9	0.02^2^
Male sex, n(%)	381(58)	41(64)	0.34^3^
BMI, kg/m^2^	26(21–32)	27(23–31)	0.16^2^
Hypertension, n(%)	251 (38%)	25 (39%)	0.89^3^
Diabetes mellitus (type 2), n(%)	151 (23%)	15 (23%)	0.93^3^
Hypercholesterolemia, n(%)	182(28)	17(26)	0.73^3^
Current smoking, n(%)	75(11)	13(20)	0.04^3^
NYHA ≥ III, n(%)	193(30)	26(31)	0.16^3^
Heart rate (bpm)	69.5 ± 13.1	65.6 ± 13.6	0.10^2^
Systolic BP (mmHg)	125.7 ± 21.1	119.9 ± 21.1	0.04^2^
Diastolic BP (mmHg)	77.4 ± 12.4	74.8 ± 14.5	0.12^2^
**Symptoms**			
Dyspnea, n(%)	234(36)	18(28)	0.20^3^
Chest pain, n(%)	295(44)	18(28)	0.09^3^
Palpitations, n(%)	79(12)	9(14)	0.64^3^
Arrhythmia, n(%)	40(6)	5(8)	0.52^3^
Syncope, n(%)	11 (2)	3(5)	0.12^3^
**Medication**			
Betablockers/Ivabradine	446 (68%)	37 (58%)	0.11^3^
RAS inhibitors/Entresto	498 (76%)	43 (67%)	0.13^3^
Prednisolone	109 (17%)	11 (17%)	0.90^3^
Colchicine	58 (8.8%)	11 (17%)	0.030^3^
**Blood markers**			
Hematocrit (%)	43.0 [39.0, 45.0]	40.0 [38.0, 45.0]	0.03^2^
eGFR, ml/min/1.73 m2	78.8 [75.0, 90.0]	78.2 [67.9, 92.5]	0.14^2^
hs-CRP (mg/l)	0.2 [0.2, 0.2]	0.2 [0.2, 0.5]	< 0.001^2^
hs-TropT (ng/l)	6.0 [5.0, 10.3]	15.0 [5.8, 41.5]	< 0.001^2^
**CMR**			
LV-EF, %	47.1 ± 13.1	43.0 ± 15.7	0.02^2^
LV-EDV (index), ml/m²	96.7 ± 36.1	119.1 ± 47.3	< 0.001^2^
LV-mass (index), g/m²	66.2 ± 25.4	75.0 ± 27.4	0.01^2^
RV-EF, %	52.9 ± 12.2	46.5 ± 15.7	< 0.001^2^
Native T1 (z)	3.5 ± 4.0	7.3 ± 3.3	< 0.001^2^
Native T1 (Siemens)	1141 ± 62	1188 ± 43	< 0.001^2^
Native T1 (Philips)	1104 ± 53	1177 ± 44	< 0.001^2^
Native T2 (z)	1.1 ± 1.1	2.2 ± 0.9	< 0.001^2^
Native T2 (Siemens)	38.1 ± 3.2	41.0 ± 2.4	< 0.001^2^
Native T2 (Philips)	50.5 ± 2.1	53.2 ± 1.5	< 0.001^2^
LGE (present), n(%)	237 (36%)	41 (64%)	< 0.001^2^
**Diagnosis CMR (definitions in** ^2^ **)**			< 0.001^3^
High-grade myocardial inflammation	128 (19%)	45 (70%)	
Low-grade myocardial inflammation	68 (10%)	16 (25%)	
Cardiomyopathy	115 (17%)	2 (3.1%)	
Healed inflammation	174 (26%)	1 (1.6%)	
Normal	173 (26%)	0 (0%)	

^1^Median [IQR]; n (%); Mean ± SD

^2^One-way ANOVA

^3^Pearson’s Chi-squared test

^4^Kruskal-Wallis rank sum test

Patients who experienced events were broadly comparable to those without events with respect to baseline demographics, medication use, and cardiac volumes and mass (Table 1). In contrast, event-positive patients exhibited significantly higher inflammatory and myocardial injury markers, including hs-CRP, hs-TropT, cardiac volumes and mass, native T1, and native T2 (all *p* < 0.01), as well as lower systolic blood pressure (*p* = 0.04), reduced LVEF (*p* = 0.02), and reduced RVEF (*p* < 0.001). LGE was more prevalent among patients with events (64% vs. 38%, *p* < 0.001). Among the 158 patients (22%) with hs-TropT concentrations at or above the upper reference limit, 94 (59%) exhibited LGE, compared with 184 (33%) in the troponin-negative group. Notably, most patients experiencing MACE showed evidence of active myocardial inflammation, classified as high-grade (*n* = 45, 70%) or low-grade (*n* = 16, 25%), irrespective of LGE presence. Event rate was not different between high-grade and low-grade inflammation (Fisher’s exact *p* = 0.27).

Univariable and multivariable analyses of the primary endpoint are summarised in Table 2. In univariable Cox analyses, age, systolic blood pressure, haematocrit, C-reactive protein (CRP), hs-TropT, left and right ventricular ejection fraction (LVEF, RVEF), cardiac volumes and mass, LGE, and native T1 and T2 were associated with MACE. In multivariable Cox regression, native T2, indexed left ventricular enddiastolic volume, hs-TropT, and the presence of LGE remained independent predictors of MACE (model χ² = 81.64, *p* < 0.001). Native T1 was removed during multicollinearity assessment. Kaplan–Meier curves illustrate associations with MACE for individual metrics (native T1, native T2, LGE, hs-TropT) and for the CMR-based definition of myocardial inflammation (Fig. 1). Patients with active myocardial inflammation (high- or low-grade) had the highest mid-term event risk (*p* < 0.001).

**Table 2:** Univariable and multivariable Cox regression analyses for the MACE endpoint. Abbreviations as in Table 1. HR, hazard ratio; CI, confidence interval

Univariable Analysis		MACE				
Characteristic		OR		95% CI		p-value
Age (years)		1.02		1.00, 1.04		0.02
Male Sex (n)		1.30		0.77, 2.24		0.30
Heart Rate (ms)		0.98		0.95, 1.00		0.11
Systolic Blood Pressure (mmHg)		0.99		0.98, 1.00		0.04
Log_Hematocrit		0.94		0.90, 1.00		0.03
Log-hsTropT		4.52		2.74, 7.48		< 0.001
Log-CRP		2.66		1.43, 4.78		0.001
Log-eGFR		0.99		0.98, 1.00		0.14
LV-EF, %		0.98		0.96, 1.00		0.02
LV-EDV (index), ml/m²		1.01		1.01, 1.02		< 0.001
LV-mass (index), g/m²		1.01		1.00, 1.02		0.01
RV-EF, %		0.96		0.95, 0.98		< 0.001
LGE (present), n(%)		3.17		1.87, 5.48		< 0.001
Native T1(z)		1.21		1.14, 1.28		< 0.001
Native T2(z)		2.32		1.85, 2.95		< 0.001
OR= Odds Ratio; CI = Confidence Interval						

Multivariate Analysis	MACE					
Characteristic	OR^1^		95% CI^1^		p-value	
Log-hsTropT	2.19		1.20, 3.95		0.010	
LV-EDV (index), ml/m²	1.01		1.00, 1.01		0.003	
LGE present	2.06		1.15, 3.73		0.016	
Native T2(z)	2.00		1.56, 2.58		< 0.001	
^1^OR = Odds Ratio, CI = Confidence Interval						

Model χ²(4) = 81.64, *p* < 0.001

Removed during backward elimination: Native T1 (z), RVEF (%)

**Fig. 2:**
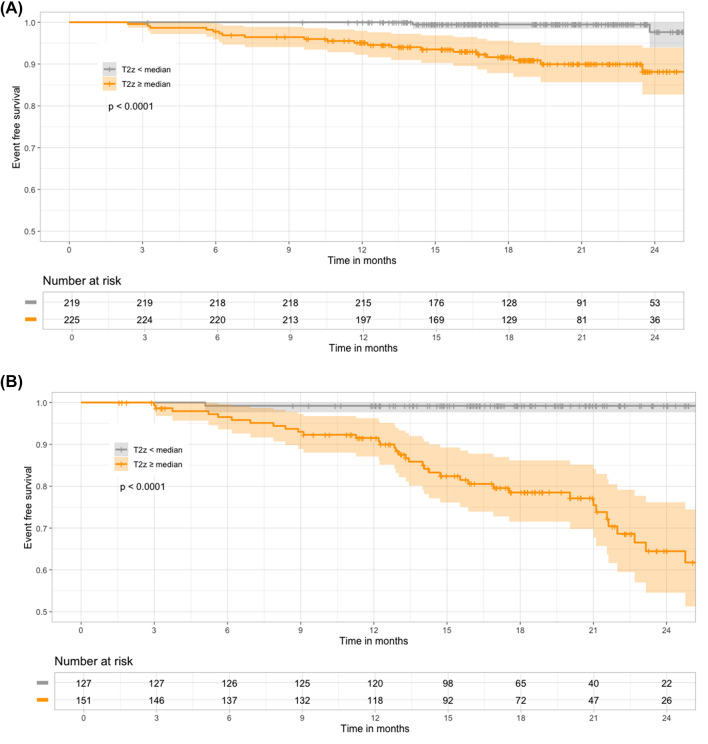

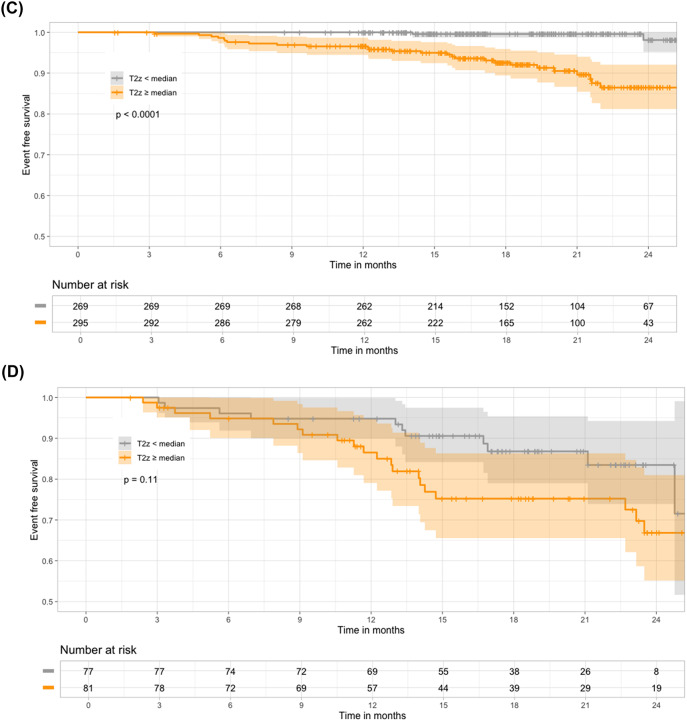
Kaplan–Meier curves illustrating the prognostic value of native T2 for MACE in patients stratified by absence (2 A) or presence (2B) of LGE, and by negative or positive troponin status. **(A)** Native T2 in patients without LGE (χ² = 17.27, *p* < 0.001). **(B)** Native T2 in present LGE (χ 33.98, *p* < 0.001). **(C)** Native T2 in negative troponin (χ 20.70, *p* < 0.001). **(D)** Native T2 in positive troponin (χ 2.59, *p* = 0.11)

Further stratification by LGE and troponin status clarified the relative contribution of each marker to event risk (Fig. 2; Supplementary Tables). T2 mapping retained prognostic significance in patients without LGE (χ² = 17.27; median z-score 17.27 vs. 30.98 in LGE-positive patients; *p* < 0.001). By contrast, T2 lost prognostic significance in patients with elevated hs-TropT (troponin-negative: χ² = 20.70, *p* < 0.001; troponin-positive: χ² = 2.59, *p* = 0.11).

## Discussion

We demonstrate that myocardial oedema, injury, and scar—assessed via T2 mapping, high-sensitivity troponin T and non-ischaemic LGE, respectively—are each independently associated with mid-term outcomes in patients with clinically suspected myocardial inflammation. Together, these findings highlight the prognostic value of multiparametric CMR and underscore the clinical utility of advanced tissue characterisation techniques. Future studies are needed to determine whether these imaging markers can guide therapeutic strategies and ultimately improve outcomes.

Our findings are derived from a large, prospective, multicentre cohort of patients referred to tertiary centres and undergoing standardised CMR as part of routine clinical evaluation, thereby reflecting real-world practice in this setting. Potential differences in healthcare systems and geographic locations were mitigated by harmonised imaging protocols, enhancing the robustness of practices and generalizability of our findings [3, 4]. Exploratory univariable analyses confirmed several established predictors of adverse outcomes in clinically suspected myocarditis, including age, haematocrit, troponin, CRP, eGFR, ventricular volumes and function, and presence of LGE, consistent with previous reports [20, 22, 28–33]. A prior study similarly established CRP as prognostic in acute myocarditis [34]. Beyond these established factors, our data highlight the prognostic relevance of contemporary tissue-characterization techniques, specifically T1 and T2 mapping. Among these, T2 mapping emerged as the strongest independent predictor of adverse events, both in the overall cohort and in centre-specific analyses. These findings provide mechanistic insight by emphasising the role of myocardial oedema alongside established markers of myocardial injury. To our knowledge, this is the first study to demonstrate myocardial oedema, quantified by T2 mapping, as a clinically meaningful prognostic marker in this population. Collectively, T2 mapping, troponin and LGE delineate the physiological continuum of myocardial injury, from early oedema and inflammation to necrosis and fibrosis, mirroring the recognised pathophysiological mechanisms of myocardial injury and remodelling [35, 36], and supporting their integrated use for risk stratification. Because T2 mapping exhibited the strongest association with outcomes, it reduces the likelihood of overlooking early disease stages, such as stage A and B myocarditis [14], thereby enabling detection of active inflammation in patients who may not fulfil conventional diagnostic thresholds based on troponin or LGE alone. The superiority of T1 and T2 mapping over conventional T2-weighted imaging has been repeatedly demonstrated (reviewed in^15^). Given the poor sensitivity of T2-weighted imaging for diffuse myocardial oedema, and its attendant risk of misdiagnosis, its role has been appropriately downgraded to optional in current recommendations [37]. In contrast, myocardial mapping provides objective, quantifiable tissue characterisation with improved reproducibility, thereby reducing diagnostic uncertainty [38–40].

Our study confirms the role of LGE as a powerful prognostic marker in clinically suspected myocarditis [21, 22]. Non-ischaemic myocardial LGE generally reflects regional necrosis in an infarct-like myocarditis; however, its sensitivity for diffuse inflammatory processes is limited [10]. Similarly, significant troponin release can be associated with acute necrotising myocarditis [2, 28, 41] or chronic active myocardial inflammation, and may overlap with areas of LGE, but does not necessarily co-localize [11]. Consequently, troponin elevation likely indicates a more diffuse myocardial process that may remain undetected by LGE imaging alone [42]. Within this context, myocardial mapping provides incremental prognostic value, particularly when troponin release is modest or LGE is absent [12]. By quantifying intrinsic magnetic tissue properties, mapping techniques enable objective detection of diffuse disease activity and may serve as biomarkers of treatment response [17].

Stratified analyses further clarify the differential prognostic contribution of individual CMR markers. In patients without LGE, T2 retained independent prognostic value (χ² = 17.27), demonstrating that diffuse myocardial oedema confers outcome risk independently of focal scar. In troponin-negative patients, T2 and left ventricular end-diastolic volume, but not LGE, predicted MACE (χ² = 20.70, *p* < 0.001), indicating that oedema-based tissue characterisation captures risk not reflected by biomarker release at the time of testing. In troponin-positive patients, T2 did not provide independent prognostic value (χ² = 2.59, *p* = 0.11). This pattern is expected: in the context of significant troponin release, active myocardial injury and oedema reflect the same underlying inflammatory process, and their prognostic signals overlap rather than complement each other. The absence of incremental T2 contribution in troponin-positive patients is therefore mechanistically consistent and does not indicate a limitation of T2 mapping.

The strong prognostic value of myocardial mapping observed in this study supports the central role of diffuse myocardial inflammation in adverse cardiac remodelling and poor clinical outcomes [43]. These results are consistent with previous reports demonstrating substantial morbidity attributable to myocardial inflammation. Notably, T2 mapping and troponin outperformed traditional markers of structural heart disease, such as LVEF, in predicting events, challenging both the primacy of LVEF in universal cardiovascular risk stratification and the assumption that clinically significant myocarditis requires significant systolic dysfunction. The SD-based thresholds used to distinguish high-grade from low-grade active inflammation were derived from diagnostic validation studies against healthy reference ranges and were not designed as prognostic thresholds. Both grades of active inflammation conferred significantly elevated MACE risk relative to inactive CMR categories; the study was not powered to discriminate outcome risk between low-grade and high-grade subgroups. Reliance on tissue-characterisation markers therefore confers greater specificity, facilitating earlier detection of myocardial inflammation, potentially before the onset of HF. Together, these insights suggest an opportunity to shift current clinical management paradigms [44, 45].

## Limitations

Several limitations should be acknowledged. First, the cohort was derived from tertiary referral centres, which may limit extrapolation to outpatient populations. Second, the findings have not been validated in paediatric patients (< 18 years). Widespread implementation of myocardial mapping requires standardised acquisition protocols and rigorous quality control. Although standardised research sequences and centralised post-processing were employed, residual technical variability—including artefacts, physiological variation, practice heterogeneity, and technological drift—may affect external validity. Qualitative LGE assessment was sufficient for the present analysis; however, quantitative scar burden may further refine risk stratification, although its routine clinical implementation remains challenging. The absence of a centralised independent endpoint committee in adjudication of clinical events, across sites cannot exclude residual misclassification risk. Biomarker sampling and CMR acquisition followed local clinical workflow rather than a mandated study protocol; heterogeneity in timing relative to symptom onset may dilute biomarker–outcome associations. Higher colchicine use among patients who experienced MACE likely reflects confounding by indication, as clinicians selectively treated those with more severe or refractory inflammation; a causal interpretation should be avoided. Residual confounding by unmeasured clinical variables cannot be excluded.

## Conclusions

In this prospective multicentre cohort of patients with clinically suspected myocardial inflammation, T2 mapping, troponin T, and non-ischaemic LGE were each independently associated with mid-term adverse cardiovascular outcomes, with T2 mapping demonstrating the strongest prognostic value. These findings support the clinical utility of quantitative CMR tissue characterisation for risk stratification and provide a foundation for future trials of imaging-guided management in this population.

## Supplementary Information

Below is the link to the electronic supplementary material.

Supplementary Material 1

## Data Availability

The dataset analysed in this study was collected under ethics approvals that do not envisage public sharing of the data. Because the cohort is relatively small and carries a risk of inadvertent re-identification, the data cannot be shared openly. Reasonable access requests may be considered on a case-by-case basis, contingent upon compliance with the original ethical approvals.
