# Supplementary material for: Outcomes by cardiovascular magnetic resonance in patients with clinically suspected myocardial inflammation
Source: Int J Cardiovasc Imaging. 2026 Apr 18;42(8):1601–10. doi: 10.1007/s10554-026-03711-y (PMC13433465; doi:10.1007/s10554-026-03711-y)
Supplement: Supplementary file 1 [file 10554_2026_3711_MOESM1_ESM.docx]

**Supplementary material**

**Full title: Outcomes by cardiovascular magnetic resonance in patients with clinically suspected myocardial inflammation**

**Extended Methods**

**Endpoint definitions**

Modes of death were classified according to a modified Hinkle–Thaler system. For composite endpoints, only the first qualifying event per patient was included in the analysis.

Cardiovascular death was defined as a composite of cardiovascular mortality, including aborted or completed sudden cardiac death (SCD), heart failure (HF), stroke, thromboembolic events, or cardiac transplantation.

SCD was defined as unexpected death occurring within 1 hour of symptom onset in the absence of progressive cardiac deterioration, during sleep, or within 24 hours of last being seen alive.

Aborted SCD was diagnosed in patients who received an appropriate implantable cardioverter-defibrillator (ICD) shock for ventricular arrhythmia or who experienced a documented non-fatal episode of ventricular fibrillation or spontaneous sustained ventricular tachycardia (>30 seconds) causing haemodynamic compromise and requiring cardioversion.

HF death was defined as death associated with unstable, progressive deterioration of cardiac pump function despite active therapy.

Appropriate ICD shocks were defined as those delivered for true ventricular tachyarrhythmias (ventricular tachycardia or ventricular fibrillation).

Competing-risk analysis was performed using a cause-specific hazard model. Patients were clinically stable at the time of inclusion, with no changes in regular medication within the preceding two weeks. None of the patients was considered for left ventricular assist device (LVAD) implantation or heart transplantation at study inclusion. Patients with a history of malignancy were required to be in remission for more than 24 months or discharged from oncological follow-up.

**CMR Sequence parameters:**

1.	Cine imaging was performed using a balanced steady-state free precession sequence with parallel imaging (SENSitivity Encoding, acceleration factor 2) and retrospective ECG gating during expiratory breath-hold (TE/TR/flip angle: 1.7 ms/3.4 ms/60°; spatial resolution 1.8 × 1.8 × 8 mm). Images were acquired as a gapless short-axis stack for assessment of cardiac volumes and function, as well as single-slice long-axis views (2-, 3-, and 4-chamber).

2.	Late gadolinium enhancement (LGE) imaging was performed using gapless whole-heart short-axis coverage approximately 15 minutes after intravenous administration of 0.1 mmol/kg gadobutrol (Gadovist®, Bayer, Leverkusen, Germany). A mid-diastolic inversion-prepared two-dimensional gradient-echo sequence was used (TE/TR/flip angle: 2.0 ms/3.4 ms/25°; acquired voxel size 1.4 × 1.4 × 8 mm), with individualised inversion times to achieve optimal myocardial nulling.

3.	T1 mapping was performed using a validated variant of a balanced steady-state free precession single breath-hold modified Look-Locker inversion recovery (MOLLI) sequence. Imaging was acquired in a single mid-ventricular short-axis slice at mid-diastole prior to contrast administration (TE/TR/flip angle: 1.64 ms/3.3 ms/50°; acquired voxel size <1.2 × 1.2 × 8 mm; phase-encoding steps n = 166; 6/8 partial Fourier; 11 images corresponding to three inversion times using a non-selective 180° inversion pulse in a 3(2)3(2)5 MOLLI scheme).

4.	T2 mapping

•	St Thomas’ Hospital: A hybrid gradient- and spin-echo (GraSE) sequence combining turbo spin-echo and echo-planar imaging was used. The sequence employed a train of refocusing 180° pulses with an odd number of additional gradient echoes per spin echo (TE/TR/flip angle: 1.4 ms/2.6 ms/90°; voxel size 1.5 × 1.5 × 8 mm; SENSE acceleration factor 2; Cartesian k-space acquisition).

•	Frankfurt and Bad Nauheim: A T2-prepared steady-state free precession sequence (T2-FLASH) was used to generate three T2-weighted images with different T2 preparation times (T2prep = 0, 24, and 55 ms). Additional parameters included repetition time 3 × RR, acquisition time 7 × RR, image matrix 96 × 160, echo spacing 2.6 ms, single-shot acquisition, flip angle 40°, bandwidth 1488 Hz/pixel, and GRAPPA acceleration factor 2.

**CMR Definitions of myocardial inflammation**^1^

1.	Active myocardial inflammation (native T1 and T2 ≥2 SD above the mean of the normal reference range):

a. High-grade inflammation: native T1 ≥5 SD and T2 ≥2 SD above the mean

b. Low-grade inflammation: native T1 between ≥2 and <5 SD and T2 ≥2 SD above the mean

2.	No active inflammation (normal native T2):

a. Healed myocarditis: T1 <2 SD, T2 <2 SD, with evidence of myocardial impairment, such as reduced LVEF (≤50%), LV dilatation, and/or non-ischaemic LGE

b. Post-inflammatory cardiomyopathy: T1 ≥2 SD and T2 <2 SD

c. Normal myocardium: T1 <2 SD, T2 <2 SD, no evidence of myocardial impairment (restitutio ad integrum)

**Figure S1**

STROBE flow diagram illustrating inclusion and exclusion criteria of the study population.

**Extended Results**

**Table 1S**. Univariable and multivariable analyses in patients with and without late gadolinium enhancement (LGE).

| **LGE absent (n=444)** | **MACE** | | |  |  |
| --- | --- | --- | --- | --- | --- |
| **Characteristic** | **OR**^1^ | | | **95% CI**^1^ | **p-value** |
| Age (years) | 1.01 | | | 0.98, 1.04 | 0.4 |
| Male Sex (n) | 1.08 | | | 0.46, 2.58 | 0.9 |
| Heart Rate (ms) | 0.99 | | | 0.94, 1.03 | 0.6 |
| Log_Hematocrit | 0.91 | | | 0.84, 1.00 | 0.049 |
| Log-hsTropT | 2.44 | | | 0.89, 5.90 | 0.061 |
| Log-CRP | 2.42 | | | 0.74, 6.75 | 0.11 |
| LVEF, % | 0.97 | | | 0.94, 1.00 | 0.081 |
| LV-EDV (index), ml/m² | 1.02 | | | 1.01, 1.02 | <0.001 |
| LV-mass (index), g/m² | 1.02 | | | 1.00, 1.03 | 0.015 |
| RVEF, % | 0.96 | | | 0.93, 0.99 | 0.004 |
| T1z | 1.24 | | | 1.13, 1.38 | <0.001 |
| T2z | 2.42 | | | 1.71, 3.51 | <0.001 |
| ^1^OR = Odds Ratio, CI = Confidence Interval | | | | | |
| **Multivariable Analysis** | |  |  | |  |
| **LGE absent (n=444)** | |  |  | |  |
| LV-EDV (index), ml/m² | | 1.01 | 1.01, 1.02 | | <0.001 |
| T2z | | 2.48 | 1.72, 3.65 | | <0.001 |
| ^1^OR = Odds Ratio, CI = Confidence Interval | | | | | |
| Model χ²(2) = 34.81, p = 2.76e-08 Removed during backward elimination: Gender, Age, log-hsTropT, T1z, LVEF, LVmass(index), RVEF | | | | | |

| **LGE present (n=278)** | **MACE** | | | |  | | | | |  | |
| --- | --- | --- | --- | --- | --- | --- | --- | --- | --- | --- | --- |
| **Characteristic** | | | **OR**^1^ | | | **95% CI**^1^ | | **p-value** | | | |
| Age (years) | | | 1.02 | | | 1.00, 1.05 | | 0.066 | | | |
| Male Sex (n) | | | 1.23 | | | 0.61, 2.56 | | 0.6 | | | |
| Heart Rate (ms) | | | 0.97 | | | 0.94, 1.01 | | 0.13 | | | |
| Log_Hematocrit | | | 0.97 | | | 0.91, 1.03 | | 0.3 | | | |
| Log-hsTropT | | | 4.74 | | | 2.47, 9.41 | | <0.001 | | | |
| Log-CRP | | | 2.16 | | | 1.03, 4.39 | | 0.036 | | | |
| LVEF, % | | | 0.99 | | | 0.97, 1.01 | | 0.4 | | | |
| LV-EDV (index), ml/m² | | | 1.01 | | | 1.00, 1.01 | | 0.068 | | | |
| LV-mass (index), g/m² | | | 1.01 | | | 0.99, 1.02 | | 0.4 | | | |
| RVEF, % | | | 0.98 | | | 0.95, 1.00 | | 0.081 | | | |
| T1z | | | 1.15 | | | 1.07, 1.24 | | <0.001 | | | |
| T2z | | | 2.12 | | | 1.57, 2.92 | | <0.001 | | | |
| ^1^OR = Odds Ratio, CI = Confidence Interval | | | | | | | | | | | |
| **Multivariable Analysis** | |  | |  | | | | | | |  |
| **LGE present (n=278)** | |  | |  | | | | | | |  |
| **Characteristic** | | | **OR**^1^ | | | | **95% CI**^1^ | | **p-value** | | |
| Log-hsTropT | | | 3.23 | | | | 1.56, 6.79 | | 0.002 | | |
| T2z | | | 1.79 | | | | 1.31, 2.51 | | <0.001 | | |
| ^1^OR = Odds Ratio, CI = Confidence Interval | | | | | | | | | | | |
| Model χ²(2) = 35.5, p = 1.96e-08 Removed during backward elimination: LVmassindex, Gender, RVEF, T1z, Age, LVEF, LV-EDV (index) | | | | | | | | | | | |

**Table 2S**. Univariable and multivariable analyses in patients with and without significant troponin release (troponin-negative and troponin-positive groups).

| **Troponin Negative (n=564)** | | **MACE** | |  | |  |
| --- | --- | --- | --- | --- | --- | --- |
| **Characteristic** | | **OR**^1^ | | **95% CI**^1^ | | **p-value** |
| **Univariable Analyses** | |  | |  | |  |
| Age (years) | | 1.01 | | 0.98, 1.03 | | 0.6 |
| Male Sex (n) | | 1.05 | | 0.49, 2.35 | | 0.9 |
| Heart Rate (ms) | | 0.97 | | 0.91, 1.01 | | 0.2 |
| Log_Hematocrit | | 0.99 | | 0.91, 1.08 | | 0.8 |
| Log-hsTropT | | 2.20 | | 0.30, 17.8 | | 0.4 |
| Log-CRP | | 1.38 | | 0.40, 4.18 | | 0.6 |
| LVEF, % | | 0.97 | | 0.95, 1.00 | | 0.042 |
| LV-EDV (index), ml/m² | | 1.02 | | 1.01, 1.03 | | <0.001 |
| LV-mass (index), g/m² | | 1.01 | | 1.00, 1.03 | | 0.019 |
| RVEF, % | | 0.97 | | 0.94, 0.99 | | 0.013 |
| LGE (present), n(%) | | 1.85 | | 0.85, 3.98 | | 0.11 |
| Native T1 (z) | | 1.21 | | 1.11, 1.32 | | <0.001 |
| Native T2 (z) | | 2.53 | | 1.78, 3.69 | | <0.001 |
| ^1^OR = Odds Ratio, CI = Confidence Interval | | | | | | |
| **Multivariable Analysis** |  | |  | |  | |
| **MACE** |  | |  | |  | |
| **Characteristic** | **OR**^1^ | | **95% CI**^1^ | | **p-value** | |
| LVEDV (index), ml/m2 | 1.02 | | 1.01, 1.03 | | <0.001 | |
| Native T2 (z) | 2.51 | | 1.74, 3.74 | | <0.001 | |
| ^1^OR = Odds Ratio, CI = Confidence Interval | | | | | | |
| Model χ²(2) = 43.22, p = 4.13e-10 No variables removed during backward elimination. | | | | | | |

| **Troponin positive (n=158)** | | **MACE** |  |
| --- | --- | --- | --- |
| **Characteristic** | **OR**^1^ | **95% CI**^1^ | **p-value** |
| Age (years) | 1.02 | 0.99, 1.04 | 0.14 |
| Male Sex (n) | 1.94 | 0.90, 4.33 | 0.10 |
| Heart Rate (ms) | 0.99 | 0.95, 1.02 | 0.4 |
| Log_Hematocrit | 0.94 | 0.88, 1.01 | 0.10 |
| Log-hsTropT | 0.94 | 0.88, 1.01 | 0.10 |
| Log-CRP | 1.41 | 0.50, 3.85 | 0.5 |
| LVEF, % | 1.00 | 0.97, 1.03 | >0.9 |
| LV-EDV (index), ml/m² | 1.00 | 0.99, 1.01 | 0.8 |
| LV-mass (index), g/m² | 1.01 | 0.99, 1.02 | 0.3 |
| RVEF, % | 0.98 | 0.95, 1.00 | 0.079 |
| LGE (present), n(%) | 2.97 | 1.30, 7.46 | 0.013 |
| Native T1 (z) | 1.08 | 0.98, 1.18 | 0.12 |
| Native T2 (z) | 1.53 | 1.11, 2.15 | 0.012 |

^1^OR = Odds Ratio, CI = Confidence Interval

| **Troponin positive (n=158)** |  | |  | |  |  |  | |  |
| --- | --- | --- | --- | --- | --- | --- | --- | --- | --- |
| **Multivariable Analysis** | |  | |  | | | |  | |
| **MACE** | |  | |  | | | |  | |
| **Characteristic** | | **OR**^1^ | | **95% CI**^1^ | | | | **p-value** | |
| LGE (present), n(%) | | 2.77 | | 1.20, 7.06 | | | | 0.023 | |
| Native T2 (z) | | 1.50 | | 1.07, 2.13 | | | | 0.021 | |
| ^1^OR = Odds Ratio, CI = Confidence Interval | | | | | | | | | |
| Model χ²(2) = 12.37, p = 0.00206 Removed during backward elimination: RVEF | | | | | | | | | |

1. Zainal, H. *et al.* Comparison of diagnostic algorithms in clinically suspected viral myocarditis: Agreement between cardiovascular magnetic resonance, endomyocardial biopsy, and troponin T. *J. Cardiovasc. Magn. Reson.* **26**, 101087 (2025).
